# Improving transparency and quality assurance: Operationalising semi-automated data provenance tracking in a Trusted Research Environment

**DOI:** 10.23889/ijpds.v9i5.2539

**Published:** 2024-09-18

**Authors:** Katherine O'Sullivan, Milan Markovic, Jaroslaw Dymiter, Adrian Martin, Chinasa Odo, Helen Rowlands, Ana Ciocarlan, Katie Wilde, Arlene Casey

**Affiliations:** 1 University of Aberdeen; 2 University of Edinburgh

We present a prototype solution for improving transparency and quality assurance of the data linkage process through a data provenance dashboard designed to assist data analysts, researchers and information governance teams in authenticating and auditing data workflows within a trusted research environment (TRE). Building on prior research (Scheliga, et al., 2022), this work describes our first operationalised prototype tested in a real-world setting.

The prototype development involved four stages: (1) Co-design of interfaces with end users for provenance data collection and visualisation, producing a low-fidelity design; (2) Extension and refinement of the Safe Haven Provenance (SHP) ontology (https://tre-provenance.github.io/SHP-ontology/releases/v0.2/index-en.html); (3) Design and implementation of mechanisms for semi-automated collection of data linkage provenance using the SHP ontology; (4) Implementation and user evaluation of the dashboard.

A participatory design process with data analysts, researchers and information governance teams resulted in a low-fidelity prototype and was validated via public consultations to ensure it met public trust. The resulting prototype dashboard (https://tre-provenance.github.io/) was built as an offline desktop app to match the deployment TRE requirements. The interactive dashboard displays the data linkage information extracted from a knowledge graph described using the SHP ontology (e.g., modifications of datasets, data release) and results of rule-based validation checks (e.g., checking extracted data against researchers' specification). User evaluations confirmed the dashboard would contribute to better quality of data linkage.

This project demonstrates the next stage in advancing transparency and quality assurance within TREs by semi-automating and systematising data tracking from ingress to egress in a single tool.

